# Cross-Sectional Dimension Dependence of Electroosmotic Flow in Fractal Treelike Rectangular Microchannel Network

**DOI:** 10.3390/mi11030266

**Published:** 2020-03-04

**Authors:** Dalei Jing, Xuekuan Zhan

**Affiliations:** School of Mechanical Engineering, University of Shanghai for Science and Technology, Shanghai 200093, China; zhanxuekuan@163.com

**Keywords:** fractal treelike network, microchannel, electroosmotic flow, fluidic resistance, dimensional optimization

## Abstract

The present work theoretically and numerically studies the electroosmotic flow (EOF) within a fractal treelike rectangular microchannel network with uniform channel height. To obtain minimum EOF fluidic resistance, the microchannel cross-sectional dimensions of the fractal network are optimized. It is found that the cross-sectional dimension dependence of EOF fluidic resistance within a symmetric fractal network is only dependent on the channel width when the total channel volume is constant, and the optimal microchannel widths to reach the minimum EOF fluidic resistance satisfy the scaling law of *κ* = *N*^−1^ (where *κ* is the width ratio of the rectangular channels at two successive branching levels, *N* is the branching number); however, for the symmetric fractal network with constant total surface area, the optimal cross-sectional dimensions should simultaneously satisfy *κ* = *N*^−1^ and $H=\frac{S}{4l_{0}}\frac{1-\gamma N}{1-{\left(\gamma N\right)}^{m+1}}$ (where *H* is the channel height, *S* is the total channel surface area, *l*_0_ is the channel length at the original branching level, *γ* is the channel length ratio at two successive branching levels and *m* is the total branching level) to obtain the minimum EOF fluidic resistance. The optimal scaling laws established in present work can be used for the optimization design of the fractal rectangular microchannel network for EOF to reach maximum transport efficiency.

## 1. Introduction

Over the past decades, microfluidic devices have attracted wide scientific attentions and been widely used in fields such as biological and chemical detections, drug delivery, and micromixing [1,2,3,4]. For the microscale flow, surface charge generated at the solid wall-ionic liquid interface is a significant interfacial property to affect the microscale fluid flow [5,6,7,8,9,10]. This is because the surface charge at the solid–liquid interface can redistribute the charged ions in the ionic liquid and forms the electrical double layer (EDL) with local net charge density [5,6,7,8,9,10]. However, because of the characteristic length of the EDL known as Debye length is small and has the typical values from several nanometers to one micrometer [6,7,8], thus the effect of EDL on the macroscale flow is usually neglectable and it can only produce obvious effect on the micro/nanoscale fluid flow. When an external electric field is applied on the ionic liquid with EDL within a microchannel, the liquid will be driven by the electric field and form the electroosmotic flow (EOF) [10,11,12,13,14,15,16], which is a typical fluidic transport phenomenon over the microscale. EOF has many advantages such as no mechanical parts, easy to design, and high level of flow control, thus, it has wide applications. For example, electroosmotic micromixing can effectively enhance the micromixing process [17,18]. Electroosmotic pump is a promising device to generate pulsate free, pluglike, and high-pressure flow for microscale fluid delivery [19,20]. To understand the EOF in microfluidics, numerous studies have been performed to analyze its transport process in microchannels. For example, Herr et al. [14] analytically and experimentally studied the EOF in cylindrical capillaries with non-uniform surface charge distributions. Xuan and Li [15] studied both direct current and alternating current EOF in microchannels with arbitrary cross-sectional geometry and arbitrary distribution of wall charge. Shamloo et al. [17] numerically studied the heat transfer performance of mixed electroosmotic and pressure-driven flow in straight microchannels with asymmetrical and symmetrical surface charge distributions.

To achieve microfluidic applications like drug delivery, biological and chemical detections, micromixing and particle separation, the actual microfluidic devices is usually with complicated microchannel layout. Fractal treelike microchannel network inspired by the treelike bifurcation in nature has become a commonly used channel layout with good transport performance for microfluidic applications [21,22]. However, because of the small dimensions of the microchannel, the mass transport efficiency within the treelike microchannel network is low, thus, how to improve the fluidic transport efficiency within the microfluidic network by optimization design is important. Actually, for laminar flow, turbulent flow, convective heat transfer and heat conduction, the structural and dimensional parameters of the fractal treelike microchannel network has been optimized to improve the corresponding transport efficiency [23,24,25,26,27,28,29,30]. For the EOF, although the dimension optimization of the treelike circular microchannel network under constant total channel volume has been performed to reach maximum mass transfer efficiency [31], however, the optimization of the treelike microchannel network with rectangular cross-sectional shape for EOF to reach maximum mass transfer efficiency is still needed, because the rectangular microchannel system has much wider applications in microfluidic devices considering the limitation of manufacturing techniques.

Thus, the present work theoretically and numerically studies the EOF in the fractal treelike rectangular microchannel network, and performs the microchannel cross-sectional dimension optimization of the two fractal microchannel networks with different size limitations to obtain the minimum EOF fluidic resistance defined as the ratio of applied driven voltage and the corresponding flowrate. The primary aim of the present work is to find out the optimal scaling laws of the channel cross-section dimensions for EOF to reach maximum transport efficiency in the symmetric fractal treelike rectangular microchannel network.

## 2. Theoretical Modeling

**Figure 1:**
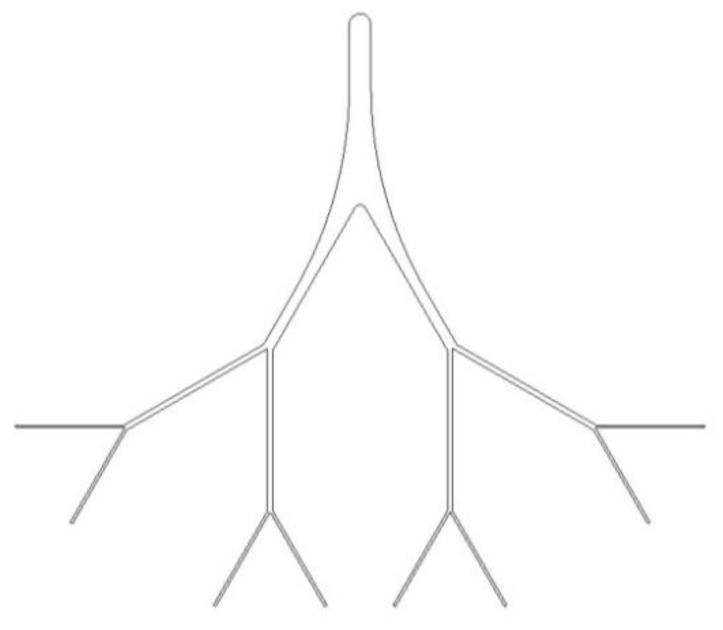
Symmetrical fractal treelike microchannel network.

In present work, a fractal treelike microchannel network with self-similarity as shown in Figure 1 is considered. Considering the easy manufacturability, the microchannel cross-sectional shape of rectangle with uniform height is chosen. To keep the symmetry of the network, every single microchannel from the original branching level is divided into *N* channels with the same channel length and channel cross-sectional dimensions at the following branching level. Furthermore, the channel dimensions at the two successive branching levels satisfy the following scaling laws.

(1) $$\{\begin{array}{c} \kappa =W_{i+1}/W_{i}\\ \gamma =\frac{l_{i+1}}{l_{i}}\\ H_{i+1}=H_{i} \end{array}\;\left(i=0,1,2\ldots \ldots ,m\right)$$

where *γ* is the length ratio of the microchannels at the (*i*+1)th level and *i*th level, *κ* is the width ratio of the rectangular microchannels at the (*i*+1)th and *i*th branching levels, *l_i_* and *l_i_*_+1_ are the microchannel lengths at the *i*th and the (*i*+1)th branching levels, *W_i_* and *W_i_*_+1_ are the widths of rectangle microchannels at the *i*th and (*i*+1)th branching levels, *H_i_* and *H_i_*_+1_ are the heights of rectangle microchannels at the *i*th and (*i*+1)th branching levels, *i* is the branching level index starting from 0 to *m*, and *m* is the maximum branching level of the treelike network.

To simplify the modeling, fully developed incompressible laminar EOF is assumed within the whole microchannel network. Considering EOF is the liquid motion driven by an external driven voltage along the microchannel, here, the following EOF fluidic resistance *R_H_* is introduced to characterize EOF mass transport efficiency in a single microchannel based on the electric circuit analogy [31,32].

(2) $$R_{H}=\frac{\Delta V_{p}}{Q}$$

where Δ*V_p_* is the voltage difference along the microchannel, and *Q* is the EOF flowrate under the given voltage. Obviously, the EOF fluidic resistance refers to the driven voltage consumption per unit flowrate, and a smaller fluidic resistance means a larger EOF flowrate under the given voltage difference, that is a larger EOF mass transfer efficiency. The corresponding optimal objective in present work is the minimum EOF fluidic resistance within the fractal microchannel network under certain size limitation. 

Assuming uniform zeta potential at all the channel walls and the microchannel cross-sectional dimensions at each branching level is much larger than EDL Debye length, the EOF fluidic resistance *R_H_* in a single microchannel can be further given as follows based on the EOF Helmholtz-Smoluchowski velocity [10].

(3) $$R_{H}=\frac{\Delta V_{p}}{vA}=\frac{\mu l}{\varepsilon \zeta A}=\frac{\mu l}{\varepsilon \zeta HW}$$

where *v* is the EOF Helmholtz-Smoluchowski velocity, *A* is the microchannel cross-sectional area, *ε* is the liquid permittivity, *ζ* is the zeta potential at the channel wall, *μ* is the liquid dynamic viscosity, *l* is the channel length, *H* is the height of the rectangular channel and *W* is the width of the rectangular channel.

Then, the total EOF fluidic resistance within the network with *m* branching level and *N* branching number can be given as follows based on the series-parallel connection of the network and the electric circuit analogy [32].

(4) $$R_{H\_total}=\overset{m}{\underset{i=0}{\sum }}\frac{R_{H,i}}{N^{i}}=\overset{m}{\underset{i=0}{\sum }}\frac{\mu l_{i}}{\varepsilon \zeta A_{i}N^{i}}=\frac{\mu l_{0}}{\varepsilon \zeta HW_{0}}\frac{1-{\left(\frac{\gamma }{N\kappa }\right)}^{m+1}}{1-\frac{\gamma }{N\kappa }}$$

where *R_H,i_* is the EOF fluidic resistance in each microchannel at the *i*th branching level, and *A_i_* is the channel cross-sectional area at the *i*th branching level.

To perform the microchannel cross-sectional dimension optimization, some size constraint (e.g., total channel volume, total channel surface area, and total channel length) should be introduced [25,29]. Here, two different size limitations are applied. One is the widely-used size limitation for the optimization of fractal branching system [25,29], constant total channel volume, which can be used as a measure of fluidic consumption in the microfluidic systems. The other one is the constant total channel surface area, which is the effective convective heat transfer area for the application and optimization of microchannel heat sink. Additionally, the total channel surface area can be used as a measure of the mass of the microfluidic system because mass = surface area × wall thickness × density, which should be limited in applications needing lightweight design [29].

(1) Volume Limitation

Under the volume limitation, the constant total channel volume can be given as,

(5) $$V=\overset{m}{\underset{i=0}{\sum }}V_{i}N^{i}=\overset{m}{\underset{i=0}{\sum }}A_{i}l_{i}N^{i}=HW_{0}l_{0}\frac{1-{\left(N\kappa \gamma \right)}^{m+1}}{1-N\kappa \gamma }$$

where *V* is the total channel volume, and *V_i_* is the volume of each channel at *i*th branching level.

To perform the channel cross-sectional dimension optimization, the structural and dimensional parameters of the microchannel network including *l*_0_, *γ*, *N,* and *m* are assumed to be the given parameters, then the rectangular channel width at the 0th branching level can be given as,

(6) $$W_{0}=\frac{4V}{Hl_{0}}\frac{1-N\kappa \gamma }{1-{\left(N\kappa \gamma \right)}^{m+1}}$$

Introducing Equation (6) into Equation (4), the total EOF fluidic resistance in the symmetric treelike rectangular microchannel network with volume limitation can be given as,

(7) $$R_{H\_total}=\frac{\mu l_{0}{}^{2}}{\varepsilon \zeta V}\frac{1-{\left(N\kappa \gamma \right)}^{m+1}}{1-N\kappa \gamma }\frac{1-{\left(\frac{\gamma }{N\kappa }\right)}^{m+1}}{1-\frac{\gamma }{N\kappa }}$$

From Equation (7), it can be found that the total EOF fluidic resistance under volume limitation is dependent on the channel width ratio *κ* but is independent on the channel height. By calculating $\frac{\partial R_{H\_total}}{\partial \kappa }=0$, the optimal microchannel cross-sectional dimensional relation under volume limitation where the total EOF fluidic resistance is minimum, that is maximum EOF mass transport efficiency can be theoretically obtained as follows.

(8) $$\kappa_{O\_V}=N^{-1}$$

where the subscript *O_V* means the optimal value at the volume limitation.

(2) Surface Area Limitation

Similarly, under the surface area limitation, the constant total channel surface area can be given as,

(9) $$S=\overset{m}{\underset{i=0}{\sum }}S_{i}N^{i}=2Hl_{0}\frac{1-{\left(N\gamma \right)}^{m+1}}{1-N\gamma }+2W_{0}l_{0}\frac{1-{\left(N\kappa \gamma \right)}^{m+1}}{1-N\kappa \gamma }$$

where *S* is the total channel surface area, and *S_i_* is the surface area of each channel at *i*th branching level.

With the given *l*_0_, *γ*, *N,* and *m*, the rectangular channel width at the 0th branching level can be given as,

(10) $$W_{0}=\frac{S-2Hl_{0}\frac{1-{\left(N\gamma \right)}^{m+1}}{1-N\gamma }}{2l_{0}\frac{1-{\left(N\kappa \gamma \right)}^{m+1}}{1-N\kappa \gamma }}$$

Introducing Equation (10) into Equation (4), the total EOF fluidic resistance in the treelike microchannel network with surface area limitation can be given as,

(11) $$R_{H\_total}=\frac{2\mu l_{0}{}^{2}}{\varepsilon \zeta H\left[S-2Hl_{0}\frac{1-{\left(N\gamma \right)}^{m+1}}{1-N\gamma }\right]}\frac{1-{\left(N\kappa \gamma \right)}^{m+1}}{1-N\kappa \gamma }\frac{1-{\left(\frac{\gamma }{N\kappa }\right)}^{m+1}}{1-\frac{\gamma }{N\kappa }}$$

Equation (11) shows that the total EOF fluidic resistance in the treelike rectangular microchannel network under surface area limitation is dependent on both the channel width ratio *κ* and the channel height *H*, which is different from the treelike rectangular microchannel network under volume limitation. By calculating $\{\begin{array}{c} \frac{\partial R_{H\_total}}{\partial \kappa }=0\\ \frac{\partial R_{H\_total}}{\partial H}=0 \end{array}$, the optimal microchannel cross-sectional dimensions under surface area limitation at where the EOF mass transport efficiency is maximum can be theoretically obtained as follows.

(12) $$\{\begin{array}{c} \kappa_{O\_S}=N^{-1}\;\;\;\;\;\;\\ H_{O\_S}=\frac{S}{4l_{0}}\frac{1-N\gamma }{1-{\left(N\gamma \right)}^{m+1}} \end{array}$$

where the subscript *O_S* means the optimal value at the surface area limitation.

## 3. Numerical Simulation

To validate the correctness of the above theoretical analysis, a numerical study is further performed. Considering an elemental Y-shaped bifurcation is the minimum unit to generate the symmetric treelike microchannel network, thus, the symmetric Y-shaped elemental microchannel bifurcations with one parent microchannel and two daughter microchannels are designed as the simulation model. The total channel volume of *V* = 29,000,000 μm^3^ is set for the volume limitation, and the total channel surface area of *S* = 2,112,620 μm^2^ is set for the surface area limitation. Further, the channel lengths at the two branching levels are set to be constant as *l*_0_ = 4000 μm and *l*_1_ = 2828.44 μm, and the branching angle is chosen as 60 degree.

When an external electric field is applied in the ionic liquid with EDL within the Y-shaped microchannel bifurcation, an electric body force will be exerted on the liquid to generate the EOF. In present numerical simulation, three dimensional, steady state, incompressible, and laminar liquid flow is assumed. Thus, the EOF can be governed by the modified Navier–Stokes equation with electrical body force as follows [33].

(13) $$\{\begin{array}{c} \nabla \cdot v=0\\ \rho \left(v\cdot \nabla v\right)=-\nabla p+\nabla \cdot \left(\mu \nabla v\right)+\rho_{e}E\; \end{array}$$

where *v* is the EOF velocity field, *ρ* is the liquid density, *p* is the pressure, *ρ_e_* is the net charge density within the EDL and *E* is the electric field, which is related to the electric potential as [33],

(14) E=∇Φ

where *Φ* is the electrical potential, which can be simplified as the linear superposition of the applied electric potential *φ* and the electric potential *ψ* within the EDL [33]

(15) Φ=φ+ψ

The applied electric potential is governed by [33],

(16) $$\nabla^{2}\varphi =0$$

And the electric potential within the EDL is given by [33],

(17) $$\nabla^{2}\psi =-\frac{\rho_{e}}{\varepsilon }=\frac{2n_{0}ze}{\varepsilon }\sin h\left(\frac{ze\psi }{k_{b}T}\right)$$

where *n*_0_ is the bulk ionic concentration of the liquid, *z* is the chemical valence of the liquid, *e* is the elementary charge, *k_b_* is the Boltzmann constant, and *T* is the absolute temperature of the liquid. 

To perform the numerical simulation, no-slip velocity boundary condition and uniform zeta potential of −100 mV are assumed at all the channel walls. The inlet pressure and outlet pressure are set to be zero to keep the pure EOF. The inlet voltage is set to be 300 V and the outlet voltage is zero to apply the external electric field. With these boundary and initial conditions, the EOF in the elemental Y-shaped microchannel bifurcation is numerically studied using commercial software COMSOL. Deionized water with density *ρ* = 996 kg/m^3^, dynamic viscosity *μ* = 0.001 Pa·s, and permittivity *ε* = 70.8 F/m is chosen as the liquid considering its advantages including low-cost, pollution-free, easy-to-obtain and sufficient literature data of zeta potential up to hundreds of millivolt different solid substrates [34]. 

## 4. Results and Discussion

### 4.1. Models Validation

**Figure 2:**
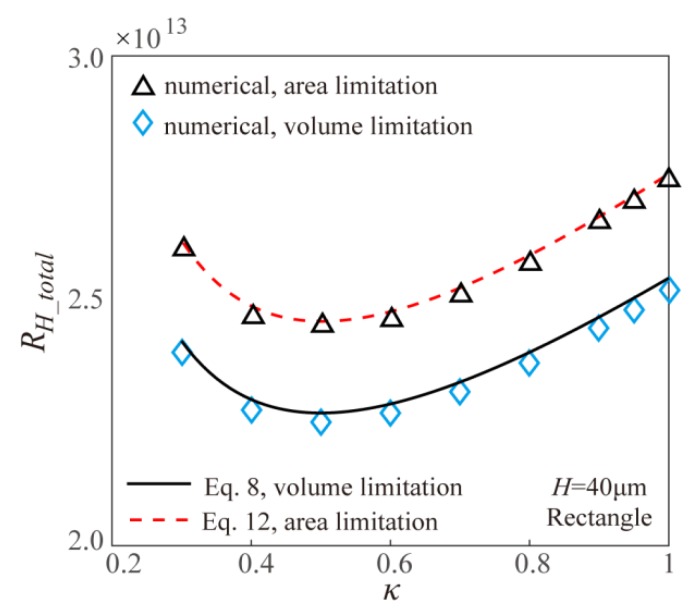
Comparison of theoretical and numerical results of electroosmotic flow (EOF) fluidic resistance within two Y-shaped microchannel bifurcations with different limitations.

To validate the correctness of both the theoretical models and the numerical simulation, Figure 2 gives the comparison of theoretical and numerical results of fluidic resistance of EOF within two Y-shaped microchannel bifurcations with uniform channel height of 40 μm both for volume limitation and surface area limitation. It can be found from Figure 2 that the theoretical result and numerical result are in good agreement with each other, which validates the correctness of models established in the present work.

**Figure 3:**
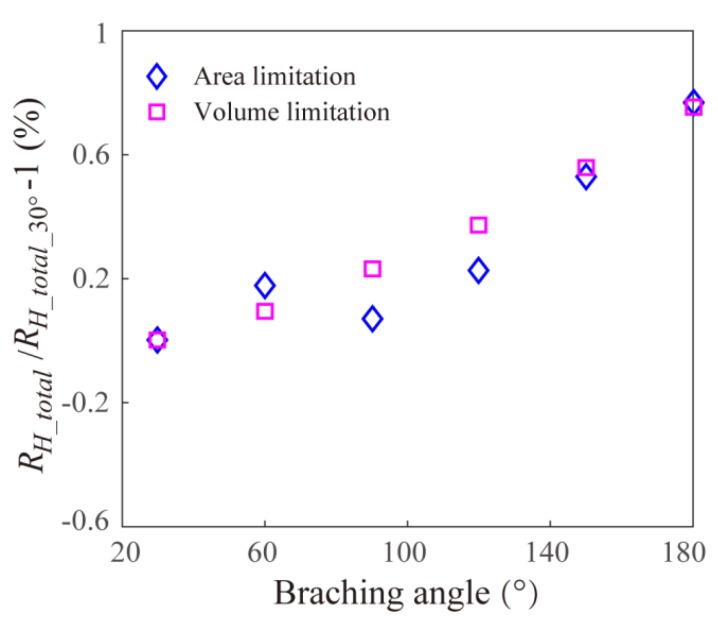
Effect of branching angle on the total fluidic resistance of EOF within two Y-shaped microchannel bifurcations with different limitations.

Furthermore, considering the branching angle is an important parameter to influence the EOF within the Y-shaped microchannel bifurcation, Figure 3 gives the numerical results of effect of branching angle on the total EOF fluidic resistance within two Y-shaped microchannel bifurcations with different limitations. It can be found that the effect of branching angle on the fluidic resistance is small enough to be neglected. This is because for the present parameter setup, the channel length is much larger than the channel cross-sectional dimensions, thus, the effect of branching angle on the EOF can be neglected.

### 4.2. Volume Limitation

**Figure 4:**
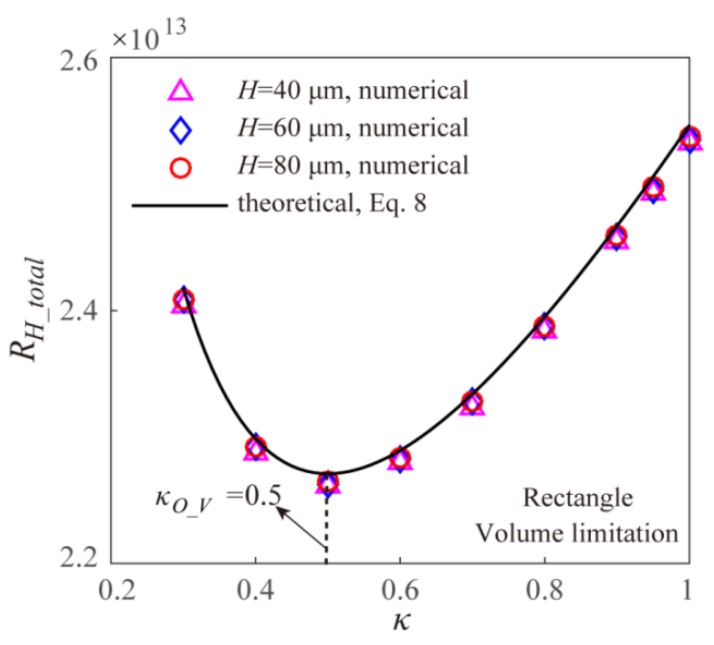
Effect of width ratio on the total EOF fluidic resistance in the Y-shaped elemental rectangular microchannel bifurcation with different channel height under the volume limitation.

Based on the theoretical models and the numerical simulation, Figure 4 gives the effect of width ratio *κ* on the EOF fluidic resistance in the Y-shaped rectangular microchannel bifurcations with different channel heights under the volume limitation. The theoretical results and the numerical results are well consistent with each other. From Figure 4, it can be found that under the volume limitation, the EOF fluidic resistance shows a decreasing-to-increasing trend with the width ratio *κ*, and the optimal width ratio to obtain the maximum EOF mass transport efficiency is 0.5, which is consistent with the theoretical result in Equation (8). When adjusting the channel height under the volume limitation, the total EOF fluidic resistance keeps constant, that is the EOF fluidic resistance is independent on the channel height, which is consistent with the theoretical model of Equation (7). This is because under the volume limitation, the rectangular channel cross-sectional area *A*(=*HW*) does not change with the unique variable of channel height *H* based on Equation (6). Further, the EOF Helmholtz-Smoluchowski velocity is not related to the rectangular channel height and width under the assumption of larger microchannel hydraulic diameter than the EDL Debye length. Thus, the EOF flowrate *Q* (= *vA*) keeps constant with the changing channel height under given applied electric field, and the corresponding total EOF fluidic resistance is independent of the channel height as shown in Figure 4.

**Figure 5:**
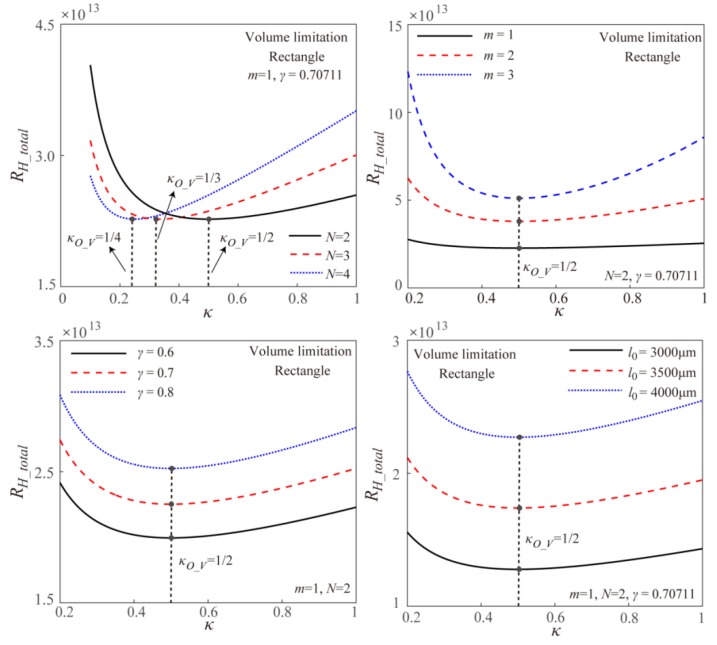
Effects of the branching number *N*, the branching level *m*, the channel length ratio *γ,* and the channel length *l*_0_ at the original branching level on the EOF fluidic resistance in the symmetric fractal rectangular microchannel network under volume limitation.

Further, Figure 5 gives the effects of the branching number *N*, the branching level *m*, the channel length ratio *γ,* and the channel length *l*_0_ at the original branching level on the EOF fluidic resistance in the symmetric fractal rectangular microchannel network under volume limitation on the basis of Equation (7). From Figure 5, it can be found that the branching number *N* is the only parameter to influence the optimal width ratio as shown in Equation (8). Furthermore, the total EOF fluidic resistance in the network under the volume limitation increases with increasing branching level *m*, increasing length ratio *γ,* and increasing original channel length *l*_0_. This is because the increasing *m*, *γ,* and *l*_0_ reduce the rectangular channel cross-sectional area, and further result in the decreasing EOF flowrate and increase the corresponding fluidic resistance.

### 4.3. Surface Area Limitation

**Figure 6:**
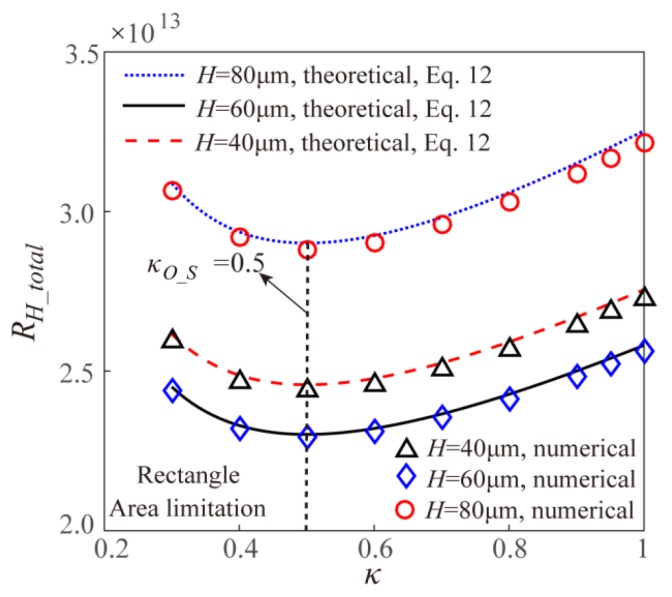
Effect of width ratio *κ* on the total EOF fluidic resistance in the Y-shaped elemental rectangular microchannel bifurcation with different channel height under the surface area limitation.

Figure 6 gives the theoretical and numerical results on the effect of width ratio *κ* on the EOF fluidic resistance in the Y-shaped elemental rectangular microchannel bifurcations with different channel heights under the surface area limitation. It can be found that the theoretical results and the numerical results are well consistent with each other. Further, the EOF fluidic resistance for the case of surface area limitation shows an increasing after decreasing trend with the increasing width ratio *κ*. For the Y-shaped rectangular microchannel bifurcations with different channel heights under the surface area limitation, their optimal width ratios are always 0.5 as shown in Figure 6, which is consistent with the theoretical optimal width ratio in Equation (12) and this means that the optimal width ratio for the symmetric fractal rectangular microchannel network under the surface area limitation is independent of the channel height. However, being different from the case of volume limitation, the channel height can affect the total EOF fluidic resistance for the cases of surface area limitation.

**Figure 7:**
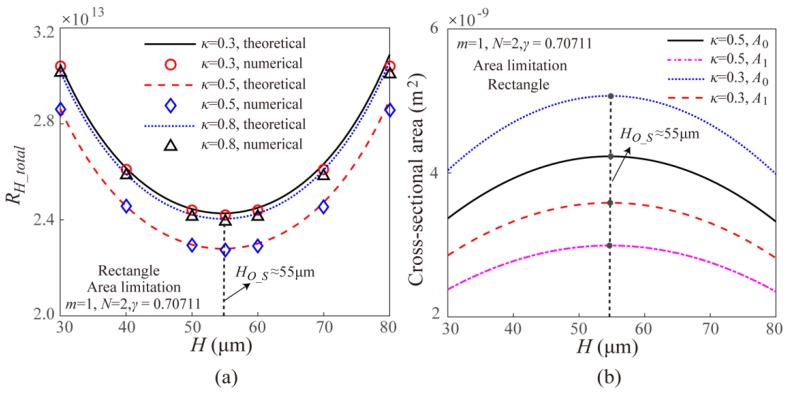
Effects of channel height on the (**a**) total EOF fluidic resistance and (**b**) channel cross-sectional areas of the Y-shaped elemental rectangular microchannel bifurcation with different channel width ratio under the surface area limitation.

Thus, Figure 7a gives the theoretical and numerical results of the effect of channel height on the total EOF fluidic resistance in the Y-shaped elemental rectangular microchannel bifurcation with different width ratio under the surface area limitation. From Figure 7a, it can be found that under the surface area limitation, the EOF fluidic resistance in the Y-shaped elemental rectangular microchannel bifurcation shows an increasing after decreasing trend with the channel height. Furthermore, the optimal channel height is independent on the width ratio *κ* and is about 55 μm for the present simulation parameters, which is consistent with the theoretical optimal channel height calculated from Equation (12). The reason for this result is that under the surface area limitation, the cross-sectional area *A* of the rectangle microchannel shows a decreasing after increasing trend with the increasing channel height as shown in Figure 7b. Further, the channel cross-sectional area *A* is maximum when the channel height is about 55 μm, which is independent on the channel width ratio *κ*. Thus, the EOF flowrate shows a decreasing after increasing trend based on *Q* = *vA*, and the corresponding EOF fluidic resistance shows an increasing after decreasing trend with the channel height and the EOF fluidic resistance is minimum when channel height is about 55 μm. Combining the results shown in Figure 6 and Figure 7, the optimal channel cross-sectional dimensions for the Y-shaped elemental rectangular microchannel bifurcation under the surface area limitation to reach a minimum EOF fluidic resistance should simultaneously satisfy the width ratio and channel height shown in Equation (12). 

**Figure 8:**
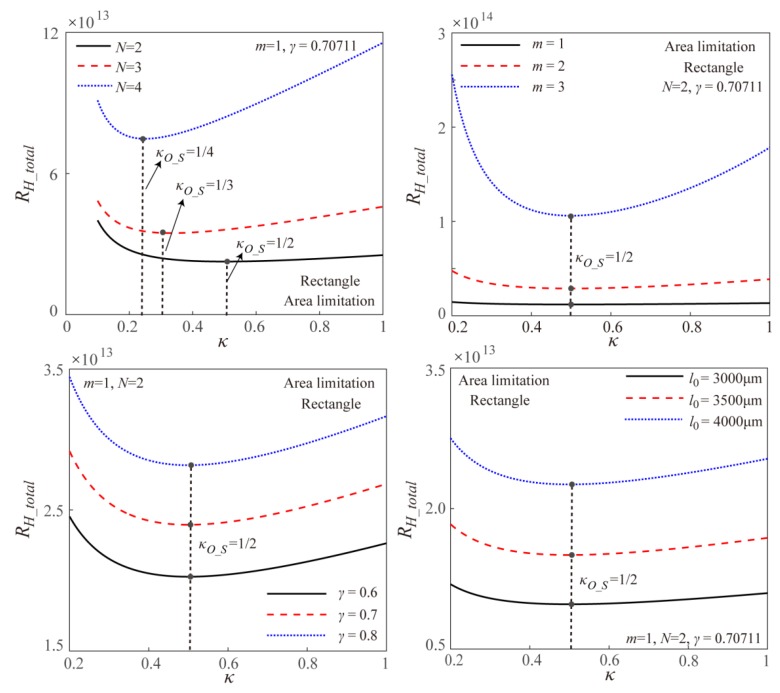
Effects of width ratio on the total EOF fluidic resistance within the symmetric fractal rectangular microchannel network with different *N*, *m*, *γ,* and *l*_0_ under surface area limitation.

Based on the theoretical model, Figure 8 gives the effects of the branching number *N*, the branching level *m*, the channel length ratio *γ* at two successive branching levels and the channel length *l*_0_ at the original branching level on the total EOF fluidic resistance in the symmetric fractal rectangular microchannel network under surface area limitation. It is found that the branching number *N* is the unique parameter to influence the optimal width ratio as the manner shown in Equation (12). Further, the total EOF fluidic resistance increases with the increase of the branching level *m*, the channel length ratio *γ* and the channel length *l*_0_. This is because the increasing *m*, *γ,* and *l*_0_ reduce the rectangular channel cross-sectional area of the rectangular microchannel network under the surface area limitation, then results in the increasing total EOF fluidic resistance as shown in Figure 8.

**Figure 9:**
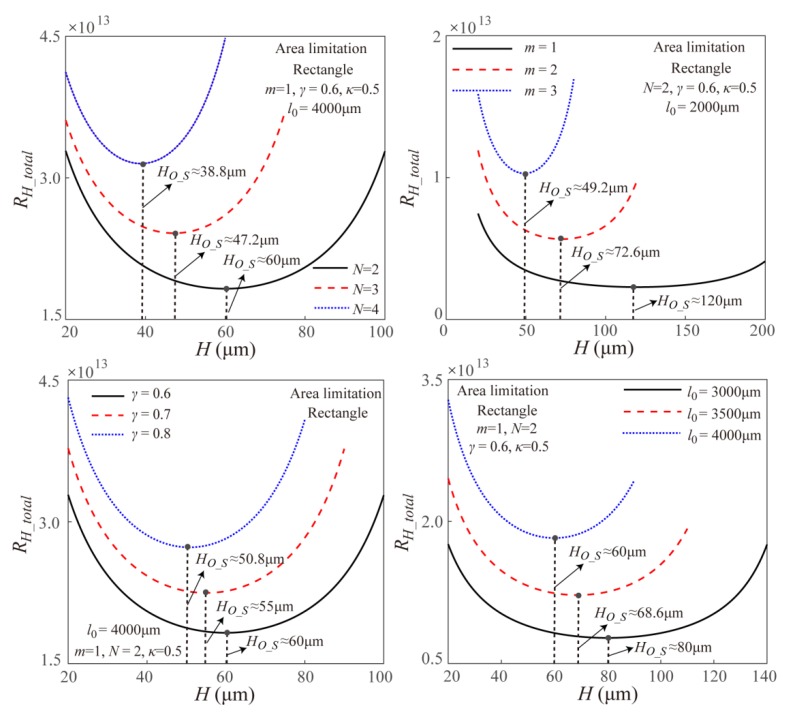
Effects of channel height on the total EOF fluidic resistance in the symmetric fractal rectangular microchannel network with different *N*, *m*, *γ,* and *l*_0_ under surface area limitation.

Figure 9 gives the effects of the channel height on the total EOF fluidic resistance in the symmetric fractal rectangular microchannel network with different *N*, *m*, *γ,* and *l*_0_ under surface area limitation. It is found that the optimal channel height increases with the decreasing branching number, the decreasing branching level *m*, the decreasing channel length ratio *γ,* and the decreasing channel length *l*_0_, and the relationship between the value of the optimal channel height with *N*, *m*, *γ,* and *l*_0_ satisfies the theoretical model in Equation (12). This further verifies the Equation (12) is correct.

In summary, for the EOF in the symmetric fractal treelike rectangular microchannel network, the optimal channel cross-sectional dimensions should satisfy the following equations to reach the maximum EOF mass transport efficiency.

$$\kappa_{O\_V}=N^{-1}\;\mathrm{for}\;\mathrm{volume}\;\mathrm{limitation}\;\{\begin{array}{c} \kappa_{O\_S}=N^{-1}\;\;\;\;\;\;\\ H_{O\_S}=\frac{S}{4l_{0}}\frac{1-N\gamma }{1-{\left(N\gamma \right)}^{m+1}} \end{array}\;\mathrm{for}\;\mathrm{surface}\;\mathrm{area}\;\mathrm{limiation}$$

## 5. Conclusions

In present work, the effects of microchannel cross-sectional dimensions on the mass transport efficiency of EOF within a symmetric fractal treelike rectangular microchannel network are theoretically and numerically studied. The present work established the optimal microchannel cross-sectional dimensions of the symmetric fractal microchannel network to reach the minimum fluidic resistance. It is found that for the microchannel network under the constant channel volume limitation, the EOF flowrate is independent of the channel height but dependent on the channel widths, and the optimal width ratio of microchannels at two successive branching levels should satisfy the scaling law of *κ* = *N*^−1^; however, for the constant surface area limitation, the optimal cross-sectional dimensions should satisfy *κ* = *N*^−1^ and $H=\frac{S}{4L_{0}}\frac{1-\gamma N}{1-{\left(\gamma N\right)}^{m+1}}$ to acheive the minimum fluidic resistance. The findings in the present work can be used for the design optimization of the fractal microchannel network for EOF to reach maximum transport efficiency under the given applied driven voltage.
